# The alternative sigma factor RpoQ regulates colony morphology, biofilm formation and motility in the fish pathogen *Aliivibrio salmonicida*

**DOI:** 10.1186/s12866-018-1258-9

**Published:** 2018-09-12

**Authors:** Miriam Khider, Nils Peder Willassen, Hilde Hansen

**Affiliations:** Norwegian Structural Biology Center and the Department of Chemistry, Faculty of Science and Technology, UiT-The Arctic University of Norway, N-9037 Tromsø, Norway

**Keywords:** *Aliivibrio salmonicida*, Sigma factors, RpoQ, Temperature, Quorum sensing, Motility, Biofilm, Overexpression

## Abstract

**Background:**

Quorum sensing (QS) is a cell-to cell communication system that bacteria use to synchronize activities as a group. LitR, the master regulator of QS in *Aliivibrio salmonicida,* was recently shown to regulate activities such as motility, rugosity and biofilm formation in a temperature dependent manner. LitR was also found to be a positive regulator of *rpoQ*. RpoQ is an alternative sigma factor belonging to the sigma −70 family. Alternative sigma factors direct gene transcription in response to environmental signals. In this work we have studied the role of RpoQ in biofilm formation, colony morphology and motility of *A. salmonicida* LFI1238.

**Results:**

The *rpoQ* gene in *A. salmonicida* LFI1238 was deleted using allelic exchange. We found that RpoQ is a strong repressor of rugose colony morphology and biofilm formation, and that it controls motility of the bacteria. We also show that overexpression of *rpoQ* in a *ΔlitR* mutant of *A. salmonicida* disrupts the biofilm produced by the *ΔlitR* mutant and decreases its motility, whereas *rpoQ* overexpression in the wild-type completely eliminates the motility.

**Conclusion:**

The present work demonstrates that the *RpoQ* sigma factor is a novel regulatory component involved in modulating motility, colony morphology and biofilm formation in the fish pathogen *A. salmonicida*. The findings also confirm that RpoQ functions downstream of the QS master regulator LitR. However further studies are needed to elucidate how LitR and RpoQ work together in controlling phenotypes related to QS in *A. salmonicida*.

**Electronic supplementary material:**

The online version of this article (10.1186/s12866-018-1258-9) contains supplementary material, which is available to authorized users.

## Background

*Aliivibrio salmonicida* belongs to the *Vibrionaceae* family, which is widely distributed in the environment, mainly in the aquatic habits. Members of this family may exist in symbiotic or pathogenic relations with their hosts [1]. According to current taxonomy, *A. salmonicida* belongs to the *Aliivibrio* genus together with its three most closely related species *Aliivibro logei*, *Aliivibrio wodanis* and *Aliivibrio fischeri* [2].

*A. salmonicida* causes cold water vibriosis or Hitra disease in farmed Atlantic salmon (*Salmo salar L*), Atlantic cod (*Gadus morhua*) and rainbow trout (*Oncorhynchus mykiss*). The disease occurs mainly during late autumn and winter seasons when the seawater temperature is below 12 °C. *A. salmonicida* is a gram-negative psychrophilic bacterium with a rod shape and nine polar flagella for motility and colonization [3–5].

Members of the *Vibrionaceae* family use quorum sensing (QS) for cell-to-cell communication to regulate gene expression in response to cell density by secretion and sensing of extracellular signals called auto-inducers (AIs). As the bacterial population density increases, AIs accumulate in the environment. When the AI concentration increases above a certain threshold, the bacteria detect this and modulate gene expression [6, 7]. N-acyl homoserine lactones (AHLs) are the major class of AIs in gram-negative bacteria, and were first described in *A. fischeri* [8, 9] and *Vibrio harveyi* [10]. The QS systems in *A. fischeri* control properties such as motility, squid colonization and bioluminescence [11–13]*. A. fischeri* has two AHL based systems, LuxI/LuxR and AinS/AinR, which are primarily responsible for regulating bioluminescence and colonization factors [14]. In addition to the LuxI/LuxR and AinS/AinR systems, *A. fischeri* has the LuxS/LuxPQ QS system [14, 15]. LuxI is responsible for the synthesis of the autoinducer N-3-(oxo-hexanoyl)-homoserine lactone (3-oxo-C6-HSL) which binds the cytoplasmic receptor LuxR. LuxR then functions as a transcription activator for the luciferase *luxICDABE* operon [16]. LuxS and AinS synthesize signal molecules which are sensed by LuxPQ and AinR, respectively. The two signal systems work in parallel and convey the signal responses to LuxU-LuxO. At low cell density when AIs are not produced, LuxPQ and AinR act as kinases and relay phosphates to LuxU, which in turns phosphorylates LuxO. Phosphorylated LuxO activates the transcription of *qrr* which binds and destabilizes the mRNA of the master QS regulator LitR [12, 15, 17]. At high cell density, the AI produced by AinS (C8-HSL) accumulates in the environment and results in dephosphorylation of LuxO. When LuxO is dephosphorylated, the *qrr* level decreases and allows LitR translation. In turn, LitR activates the transcription of *luxR* which contributes to bioluminescence [12, 13].

*A. salmonicida* has three QS systems similar to those in *A. fischeri*: LuxS/LuxPQ, LuxI/LuxR and AinS/AinR [18]*.* LuxI is responsible for the synthesis of a total of seven AHLs, while AinS synthesizes only one AHL. This AHL diversity may suggest a complex sensing system which allows more fine-tuned responses to changes in the environment [19]. *A. salmonicida* does not produce bioluminescence per se [20], but regulates activities such as virulence, motility, colony morphology, adhesion, and biofilm formation by QS in a temperature dependent manner [21, 22].

Sigma factors are essential dissociable subunits of prokaryotic RNA polymerase that control promoter recognition and transcription initiation [23, 24]. Primary sigma factors (RpoD, σ^70^ family) direct transcription from the promoters of genes required for basic cellular functions. In addition to the primary sigma factors, bacteria have a variable number of alternative sigma factors whose activities increase in response to certain environmental conditions or stress [25].

Several alternative sigma factors have been identified or predicted in vibrios and aliivibrios [26], and recently a divergent copy of a putative RpoS-like sigma factor was identified in *A. fischeri* and named RpoQ due to its activation by the AinS/AinR QS system [27]*.* RpoQ was later found to regulate bioluminescence, motility and chitinase activity in *A. fischeri* through LuxO via LitR [28]. Pfam analysis of RpoQ identified four conserved domains (σ^70^ regions) where all were significant except for region 3. Phylogenetic analysis further revealed that region 3 in RpoQ is clearly divergent from the corresponding region in RpoD and RpoS [27, 28]. This less conserved region 3 is involved in binding the core RNA polymerase and recognition of the extended −10 promoter [29]. An RpoS-like sigma factor (RpoX) lacking region 3 has been described in *Vibrio alginolyticus,* and shown to be involved in biofilm formation and stress responses [30].

*A. salmonicida* strain LFI1238 encodes an *rpoQ* homolog (*VSAL_II0319*) similar to the one in *A. fischeri* [18, 28]. In a previous study we analyzed the transcriptomes of an *A. salmonicida ΔlitR* mutant and the isogenic wild-type strain LFI1238. The *rpoQ* gene was found to be downregulated in the *ΔlitR* mutant [31] suggesting that LitR is a positive regulator of *rpoQ* in *A. salmonicida*. In the work presented here we have studied the impact of this putative RpoS-like sigma factor in *A. salmonicida* with regard to different phenotypic traits such as biofilm formation, motility and colony morphology.

## Methods

### Bacterial strains, plasmids and culture conditions

Bacterial cells and plasmids used in this study are listed in Table 1. The wild-type *A. salmonicida* LFI1238 and the constructed mutants were grown from frozen glycerol stocks on blood agar base no. 2 (Oxiod, Cambridge, United Kingdom) with a final concentration of 2.5% NaCl (wt/vol) and 5% bovine blood (BA2.5) or on Luria-Bertani agar (Difco, BD Diagnostics, Sparks, MD) with a final concentration of 2.5% NaCl (wt/vol) (LA2.5). The primary cultures (2 ml) of *A. salmonicida* and the constructed mutants were grown from single colonies in LB2.5 at 12 °C and 220 rpm for 48 h. Secondary cultures were made by diluting the primary cultures 1:20 in LB2.5 and incubated for additional 24 h, unless otherwise indicated.

**Table 1:** Bacterial strains and plasmids used in this study

Bacterial strains or plasmids	Description	Source
*A. salmonicida*		
LFI1238	Wild-type, isolated from Atlantic cod	[18]
Δ*litR*	LFI1238 containing an in-frame deletion in *litR*	[22]
Δ*rpoQ*	LFI1238 containing an in-frame deletion in *rpoQ*	This study
Δ*rpoQ*_*c*_	Δ*rpoQ* strain complemented with wild-type copy of the *rpoQ* gene*,* Cm^r^	This study
Δ*litR-rpoQ*^*−*^	Δ*litR* stain with an insertional disruption in *rpoQ*, Cm^r^	This study
LFI1238*-*pVSV102	LFI1238 carrying pVSV102, Kn^r^	This study
Δ*litR-*pVSV102	Δ*litR* carrying pVSV102, Kn^r^	This study
Δ*rpoQ-*pVSV102	Δ*rpoQ* carrying pVSV102, Kn^r^	This study
LFI1238*-*pTM214	LFI1238 carrying pTM214, Cm^r^	This study
LFI1238-*Ptrc-rpoQ*	LFI1238 carrying pTM214-*rpoQ*, Cm^r^	This study
Δ*litR-*pTM214	Δ*litR* carrying pTM214, Cm^r^	This study
Δ*litR-Ptrc-rpoQ*	Δ*litR* carrying pTM214-*rpoQ*, Cm^r^	This study
*E. coli*		
S17λpir	Donor strain for conjugation	[65]
JM109	Strain for subcloning pGEM-T constructs	[66]
DH5α	Strain for cloning	Thermo Fisher
C118λpir	Helper strain containing pEVS104	[32]
DH5αλpir	Donor strain for conjugation harboring pVSV102	[32]
PIR2	Donor strain for conjugation harboring pTM214	[33]
Plasmids		
pDM4	Suicide vector with an R6K origin, *sacBR* and Cm^r^	[35]
pNQ705	Suicide vector with an R6K origin, Cm^r^	[35]
pDM4-Δ*rpoQ*	pDM4 containing a fragment of *rpoQ* harboring an internal deletion	This study
pNQ705-*rpoQ*_*c*_	pNQ705 containing a full length *rpoQ* and flanking sequences	This study
pNQ705*-rpoQ*^*−*^	pNQ705 containing an internal 304 bp fragment of *rpoQ*	This study
pTM214	pVSV105, *Ptrc-mCherry,* Cm^r^	[33]
pVSV102	pES213, constitutive GFP, Kn^r^	[67]
pEVS104	Helper plasmid, R6K origin, RP4, *oriT, trb, tra* and Kn^r^	[32]
pTM214-*rpoQ*	pVSV105, *Ptrc-rpoQ* (a full length *rpoQ* copy)*,* Cm^r^	This study
pGEM-T	TA cloning vector, white/blue screening, Amp^r^	Promega

The *Escherichia coli* strains S17λpir*,* CC118λpir, JM109, PIR2, DH5α λpir and DH5α were cultivated in LA or LB with 1% (wt/vol) NaCl (LA1 and LB1 respectively) and incubated at 37 °C. The suicide plasmids pDM4 (GenBank: KC795686.1) and pNQ705 (GenBank: KC795685.1) were propagated in S17λpir cells. The TA plasmid vector pGEM-T was propagated in JM109 and DH5α cells. The conjugation helper pEVS104 plasmid was propagated in the *E. coli* helper strain CC118λpir [32]. The pTM214 and pVSV102 (GFP) expression plasmids were propagated in the donor strains PIR2 and DH5αλpir, respectively [32, 33]. For selection of *E. coli* transformants, chloramphenicol (final concentration 25 μg/ml) or ampicillin (final concentration 100 μg/ml) was added to the medium. The potential *A. salmonicida* transconjugants were selected either on BA2.5 or LA2.5 supplemented with 2 μg/ml of chloramphenicol or 150 μg/ml of kanamycin.

A seawater-based medium (SWT) was used for biofilm and morphology assays. The medium contains 5 g/L of bacto peptone (BD), 3 g/L of yeast extract (Sigma) and 28 g of a synthetic sea salt (Instant Ocean, Aquarium Systems) per liter. The SWT medium was solidified with 1.5% (wt/vol) agar (Fluka).

All biological assays were carried out in triplicate.

### DNA extraction, PCR and DNA sequencing

DNA extraction, recombinant DNA techniques and transformations were performed according to standard protocols [34]. Restriction digestion, ligation, genomic DNA extraction and plasmid purification were performed as recommended by the manufacturers (NEB Biolabs, Sigma and Promega). PCR was performed using Phusion polymerase (NEB) or Taq polymerase master mix (WVR). DNA sequencing was performed using Big Dye (Applied Biosystems) with custom made primers synthesized by Sigma. The primers used for PCR and sequencing are listed in Table 2.

**Table 2:** The primers used in this study

Primers	Sequence (5–3’)	Source
RpoQ-A fwd	AATAACTCGAGCAAACGAATGACATGCAGACA	This study
RpoQ-B rev	ATCAATGCTGTTTCTTGGTTCTTC	This study
RpoQ-C fwd	AGAAACAGCATTGATCTAGGCCAAGATCTTCAA	This study
RpoQ-D rev	TATATACTAGTCGATCTCATTATCTTCGTAATACA	This study
RpoQ-G fwd	AGTTCAGGTGATCGTGTTA	This study
RpoQ-H rev	GATTTTGCGTATTGGTAACT	This study
RpoQ-E fwd	CTCGAGAACAGCATTGATGCTTACTCA	This study
RpoQ-F rev	ACTAGTATCCACCATACCGCGTAA	This study
pTM214-*rpoQ* fwd	TCGAGCTCAGAGGAGAAATTAAGCATGTTGAATATAGAATGTTCA	This study
pTM214-*rpoQ* rev	AGGTCGACCTAATTTAAAGCATTTCTAAA	This study
pNQ-fwd	TAACGGCAAAAGCACCGCCGGACATCA	Milton, D.
pNQ-rev	TGTACACCTTAACACTCGCCTATTGTT	Milton, D.

### Construction of *A. salmonicida* LFI1238 *ΔrpoQ* mutant and the complementary strain

The *rpoQ* gene (*VSAL_II0319*) was deleted in *A. salmonicida* by allelic exchange as previously described [22]. In brief, the pDM4-*ΔrpoQ* was constructed by fusion of two PCR products amplified from sequences downstream and upstream *rpoQ* in the genomic DNA of *A. salmonicida* LFI1238*.* The RpoQ*-*A and RpoQ-B primers were used to amplify the region upstream *rpoQ* (558 bp), and RpoQ-C and RpoQ-D primers for amplification of the region downstream *rpoQ* (729 bp). The downstream region contained the last 40 C-terminal codons of the *rpoQ* open reading frame. Primers RpoQ-B and RpoQ-C contain complementary sequences that enable fusion of the upstream and downstream PCR products by a second overlap-extension PCR. This fusion of the two PCR products results in removing 254 codons (including the start codon) from the *rpoQ* open reading frame. A’overhangs were added to the PCR product and ligated into pGEM-T, and transformed into *E. coli* JM109 competent cells. The insert (PCR overlap product) was digested from the pGEM-T plasmid using *Spe*I and *Xho*I, as restriction sites are included in RpoQ-A and RpoQ-D primers respectively. The digested overlap PCR product was then ligated into the corresponding restriction sites of the suicide vector pDM4 before being transformed directly to *E. coli* S17λpir cells. The resulting plasmid is named pDM4-*ΔrpoQ*.

The complementary strain *ΔrpoQ*_*c*_ was constructed by insertion of a full-length copy of the wild-type *rpoQ* gene into the original locus of the *ΔrpoQ.* The complete gene and flanking regions was amplified by PCR using RpoQ-A and RpoQ-D primers, digested as above, and ligated into the *Spe*I and *Xho*I restriction sites of the pNQ705. The resulting plasmid is named pNQ705-*rpoQ*_*c*_*.*

The pDM4-*ΔrpoQ* was transferred to *A. salmonicida* LFI1238, while the pNQ705-*rpoQ*_*c*_ construct was transferred to the *ΔrpoQ* mutant by bacterial conjugation mainly as described elsewhere [22, 35]. Briefly, donor cells *E. coli* S17λpir harboring the pDM4-*ΔrpoQ* or pNQ705-*rpoQ*_*c*_ were mated with their respective recipient cells (*A. salmonicida* wild-type or the *ΔrpoQ* mutant), at a 1:1 ratio. The donor cells were grown to mid-exponential phase to OD_600_ (optical density) of 0.7 and the recipient to an early stationary phase (OD_600_ 1.2) before they were harvested by centrifugation and washed twice in LB1 medium. The washed bacterial pellets were mixed and spotted onto BA2.5 agar plates. The plates were incubated at 20 °C for 6 h followed by an additional incubation for 17 h at 12 °C. The spotted cells were suspended in 2 ml LB2.5 and incubated overnight at 12 °C with agitation at 220 rpm. Potential transconjugants were selected after 5 days on BA2.5 supplemented with chloramphenicol. To complete the allelic exchange needed to generate the *ΔrpoQ* mutant, transconjugants (*A. salmonicida*-pDM4-*ΔrpoQ*), were streaked onto LA2.5 plate supplemented with 5% sucrose. Cells that are able to grow after the sucrose selection were selected based on the sensitivity to chloramphenicol. Chloramphenicol-sensitive cells were analyzed for deletion by PCR and verified by sequencing.

### Construction of the double mutant *A. salmonicida ΔlitR-rpoQ*^*−*^

Construction of *A. salmonicida* LFI1238 containing a *litR* in-frame deletion *(ΔlitR)* is described elsewhere [22]. The double mutant *ΔlitR*-*rpoQ*^*−*^ (Table 1) was constructed mainly as described by others [35]. Briefly, the pNQ705-*rpoQ*^*−*^ plasmid was constructed by cloning a (304 bp) PCR product amplified from an internal part of the *rpoQ* gene using the forward and reverse primer pair RpoQ-E and RpoQ-F (Table 2). The restrictions enzyme sites *Spe*I and *Xho*I were added to the 5’ end of the forward (RpoQ-E) and reverse (RpoQ-F) primers respectively in order to ligate the digested PCR product into the pNQ705 suicide plasmid. Hence, both the pNQ705 plasmid and the amplified PCR product were digested with *Spe*I and *Xho*I and ligated using T4 DNA ligase. The ligated construct (pNQ705-*rpoQ*^*−*^) was transformed into *E. coli* S17λpir. Next pNQ705-*rpoQ* was transferred to the *ΔlitR* mutant by bacterial conjugation as described above*.* The resulting double mutant strain was named *ΔlitR****-****rpoQ*^*−*^.

### Construction of *rpoQ* overexpression strains

A full length (882 base pairs) copy of the *A. salmonicida rpoQ* gene was amplified by PCR using the primer pair pTM214-*rpoQ* fwd and pTM214-*rpoQ* rev, containing the *SacI* and *SalI* restriction sites, respectively (Table 2). The resulting PCR product and the pTM214 expression vector (provided by Dr. Tim Miyashiro) were digested using *SacI* and *SalI* restriction enzymes. The digested PCR product was cloned downstream of the tryptophan promoter in the pTM214 expression vector, replacing the native *mCherry* gene. The construct was transformed to *E. coli* S17λpir cells and selected on LA1 plates. The resulting plasmid is referred to as pTM214-*rpoQ.*

The pTM214-*rpoQ* and pTM214 (control vector) was transferred to LFI1238 and *ΔlitR* by tri-parental mating using the conjugative helper strain CC118λpir carrying pEVS104 (helper plasmid) as described by others [32], with some modifications. Briefly, *E. coli* S17λpir harboring pTM214-*rpoQ* or PIR2 harboring pTM214 and helper strain CC118λpir carrying pEVS104 were grown to the mid-exponential phase at 37 °C. The recipient cells LFI1238 and *ΔlitR* were grown to the early stationary phase. The donor, helper and recipient cells were mated at a 1:1:1 ratio after being harvested by centrifugation for 1 min at 4 °C and washed with LB1 twice. The pelleted cells were mixed and spotted onto BA2.5 and incubated ON (overnight) at 16 °C. The spotted cells were resuspended in 2 ml LB2.5 and incubated ON at 12 °C and 220 rpm. Transconjugants were selected on plates with chloramphenicol. The resulting strains are named LFI1238-pTM214, LFI1238-*Ptrc-rpoQ, ΔlitR-*pTM214 and *ΔlitR-Ptrc-rpoQ.*

### Construction of green fluorescent *A. salmonicida* LFI1238, *ΔlitR* and *ΔrpoQ*

The pVSV102 plasmid encoding the green fluorescent protein (GFP) and kanamycin resistance was transferred from *E. coli* DH5αλpir to *A. salmonicida* LFI1238, *ΔlitR* and *ΔrpoQ* using the conjugative helper strain CC118λpir carrying pEVS104 as described above. The potential tagged strains were selected on BA2.5 after 5 days. The resulting strains were named LFI1238-pVSV102*, ΔlitR-*pVSV102 and *ΔrpoQ-*pVSV102. The GFP expression was confirmed microscopically using Nikon Eclipse TS100.

### Growth rate assay

The overnight secondary cultures were diluted to OD_600_ of 0.05 in a total volume of 60 ml SWT. The cultures were grown further in 250 ml baffled flask at 8 °C and 220 rpm. The optical density was measured every 3 h using Ultrospec 10 cell density meter (Amersham Biosciences).

### Motility assay

The motility assay was performed using soft agar plates containing 0.25% agar and 2.5% NaCl and with or without 1 mM isopropyl β-D-1-thiogalactopyranoside (IPTG). The primary cultures were diluted 1:40 and incubated overnight at 12 °C with agitation. The cultures were diluted to an OD_600_ of 0.4. Then 3 μl of each culture was spotted on the soft agar plates and incubated at 4, 8, 12, 14 and 16 °C for 5 days. The motility zones were monitored every 24 h for 5 days by measuring the diameter of the motile cells in the soft agar.

### Colony morphology and adhesion

The colony morphology assay was carried out as described previously [31, 36]. A 250 μl of each bacterial culture was harvested by centrifugation, and the pellet was re-suspended in 250 μl SWT. Then, 2 μl of each culture was spotted onto SWT agar plates, and incubated at 4, 8, 12, and 14 °C for up to 3 weeks. The colonies were viewed microscopically with Zeiss Primo Vert and photographed with AxioCam ERc5s at × 4 magnification. The same (three weeks old) colonies were also tested for their ability to adhere to the SWT agar. This was done by touching the colonies using a sterile plastic loop mainly as previously described [22], but the grading of the adherence was only recorded as “none” for smooth and creamy colonies, “weak” for slightly adherent and “strong” for colonies that were impossible to separate from the agar plate.

### Static biofilm assay

The biofilm assay was performed as previously described [31]. The overnight secondary cultures were diluted to an OD_600_ of 1.3 in LB2.5 The cultures were further diluted 1:10 in SWT and a total volume of 300 μl was added to each well in flat-bottom, non-tissue culture-treated Falcon 24-well plates (BD Bioscience). For the overexpression biofilm assay a total of 1 mM IPTG was added. The plates were incubated statically at 4, 8, 12, 14 and 16 °C, for 72 h and the biofilm was visualized using Nikon Eclipse TS100 microscope at 10× magnification and photographed with Nikon DS-5Mc.

### Phylogenic analyses and software

The amino acid sequences were aligned using ClustalW. The aligned sequences were then used to construct a neighbor-joining (NJ) tree using the MEGA version 7.0 [37]. Gaps in pairwise sequence comparison were deleted and the p-distance model was used. Bootstrap analyses with 500 replicates were conducted to provide confidence levels for the tree topology. Search for conserved sigma factor domains was performed using Pfam at EMBL-EBI (https://pfam.xfam.org/).

## Results

Our previous studies show that *A. salmonicida* LitR is involved in regulating a number of activities that may be important for host interactions [22], and by using microarray we identified a number of genes regulated by LitR [31]. The regulation of LitR on downstream genes could proceed either directly or indirectly. One of the genes found to be regulated by LitR was the *rpoQ* gene (*VSAL_II0319*)*.* We therefore sought to analyze the role of RpoQ in the different phenotypes known to be regulated by LitR and QS in *A. salmonicida*. To this end we constructed an in-frame deletion mutant (*ΔrpoQ*) of the wild-type strain LFI1238 by removing 254 of the 294 amino acids in RpoQ. A complementation mutant (*ΔrpoQ*_*c*_) was constructed to verify whether the observed phenotypes were due to the mutation of *rpoQ*. We do not expect the in-frame deletion (*ΔrpoQ*) or the insertion (*ΔrpoQc*) of *rpoQ* to have any polar effect(s) on downstream genes. However, it should be noted that this possibility cannot be excluded since the expression of the downstream genes in the operon was not analyzed in this work. Since temperature is an important factor involved in regulating AHL production and phenotypes related to QS in *A. salmonicida* [22, 31], the experiments were performed at different temperatures (4–16 °C).

### Deletion of *rpoQ* does not alter the growth of *A. salmonicida*

To analyze if the *rpoQ* mutation affected the vitality of *A. salmonicida* LFI1238, a growth curve assay was performed. The bacterial growth of all strains (LFI1238, *ΔrpoQ* and the complementary strain) was monitored in triplicate at 8 °C for 72 h. The *ΔrpoQ* mutant showed the same growth rate as the wild-type strain LFI1238 and the complementary strain *ΔrpoQ*_*c*_ (Additional file 1: Figure S1).

### RpoQ shows temperature dependent rugose colony morphology

The ability to form rugose colonies and biofilm are often correlated features in vibrios [38–40], and a rugose colony phenotype usually indicates high production of exopolysaccharides [39].

**Fig. 1:**
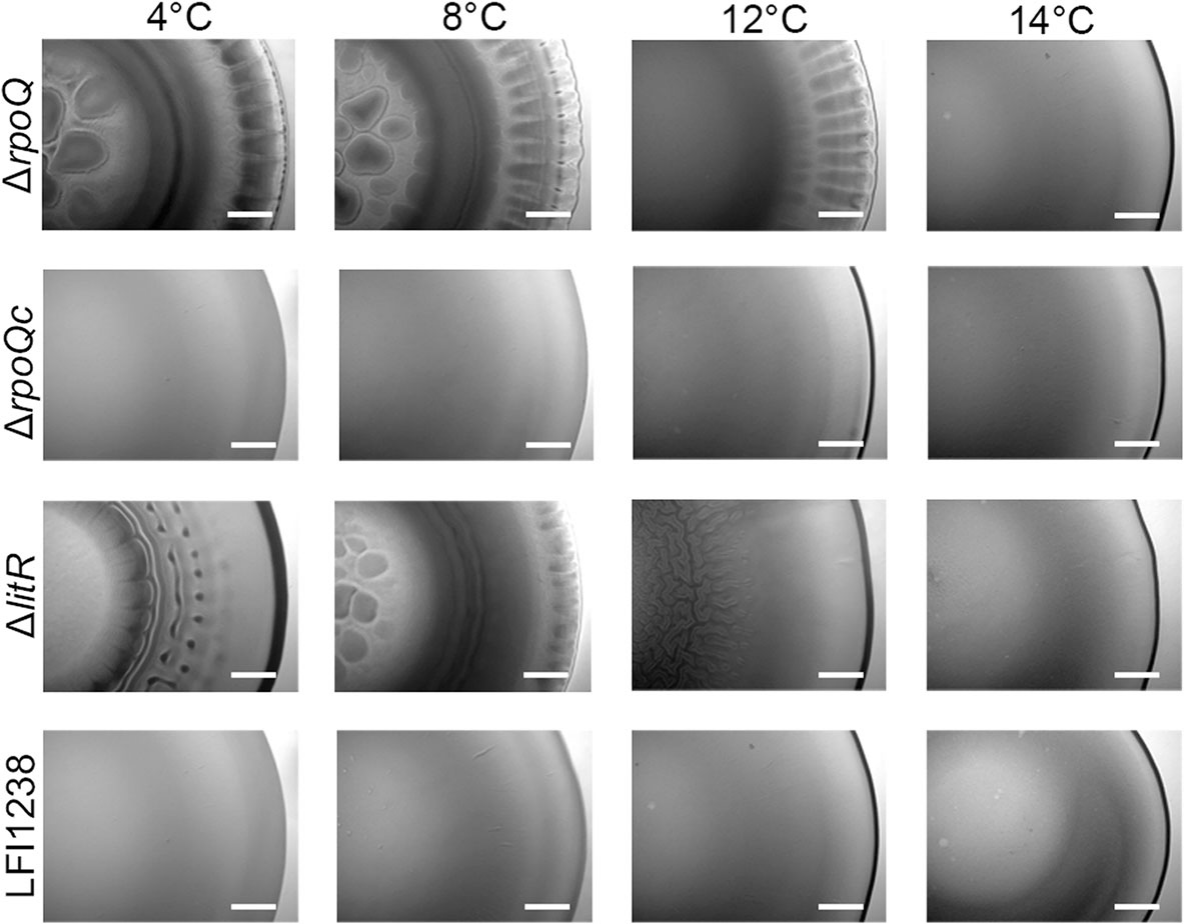
Colony morphology of Δ*rpoQ, ΔrpoQ*_*c*_*, ΔlitR* and LFI1238 at different temperatures. The colonies were allowed to form on SWT plates for 12 days at 4, 8, 12 and 14 °C. The colonies were viewed in a Zeiss Primo Vert microscope at 4× magnification. Scale bars represent 0.5 mm

To compare colony morphologies of the wild-type LFI1238, and the *ΔrpoQ* and *ΔlitR* mutants a spot colony assay was performed on SWT agar incubated at different temperatures (4 to 14 °C). The LFI1238 produced smooth colony morphology at all temperatures as previously reported [31]. The *ΔrpoQ* mutant started to form wrinkled colonies after 7 days of incubation, and at day 12 a strong rugose colony morphology with wrinkled edges was observed after growth at 4 and 8 °C (Fig. 1). When incubated at 12 °C, the *ΔrpoQ* colony remained smooth in the central part whereas the edges became wrinkled. No wrinkling was observed for *ΔrpoQ* at 14 °C. The *ΔlitR* mutant was used as positive control [31] and, compared to *ΔrpoQ,* it showed a weaker rugose colony morphology. A strong *ΔlitR* rugose colony morphology similar to the wrinkled *ΔrpoQ* colonies was observed after 3 weeks (Additional file 2: Figure S2). As previously reported the wrinkling of *ΔlitR* colonies is absent after growth at 14 °C [31].

The wrinkled colonies formed by the *ΔrpoQ* and *ΔlitR* mutants were found to be adhesive on the SWT agar, and the adhesiveness was stronger at low temperatures (4 to 8 °C). No colonies were adhesive after growth at 14 °C (Additional file 3: Table S1). The complementary strain (*ΔrpoQc*) behaved similar to the wild-type and produced non-adhesive, smooth and creamy colonies at all temperatures.

### RpoQ is involved in biofilm formation

**Fig. 2:**
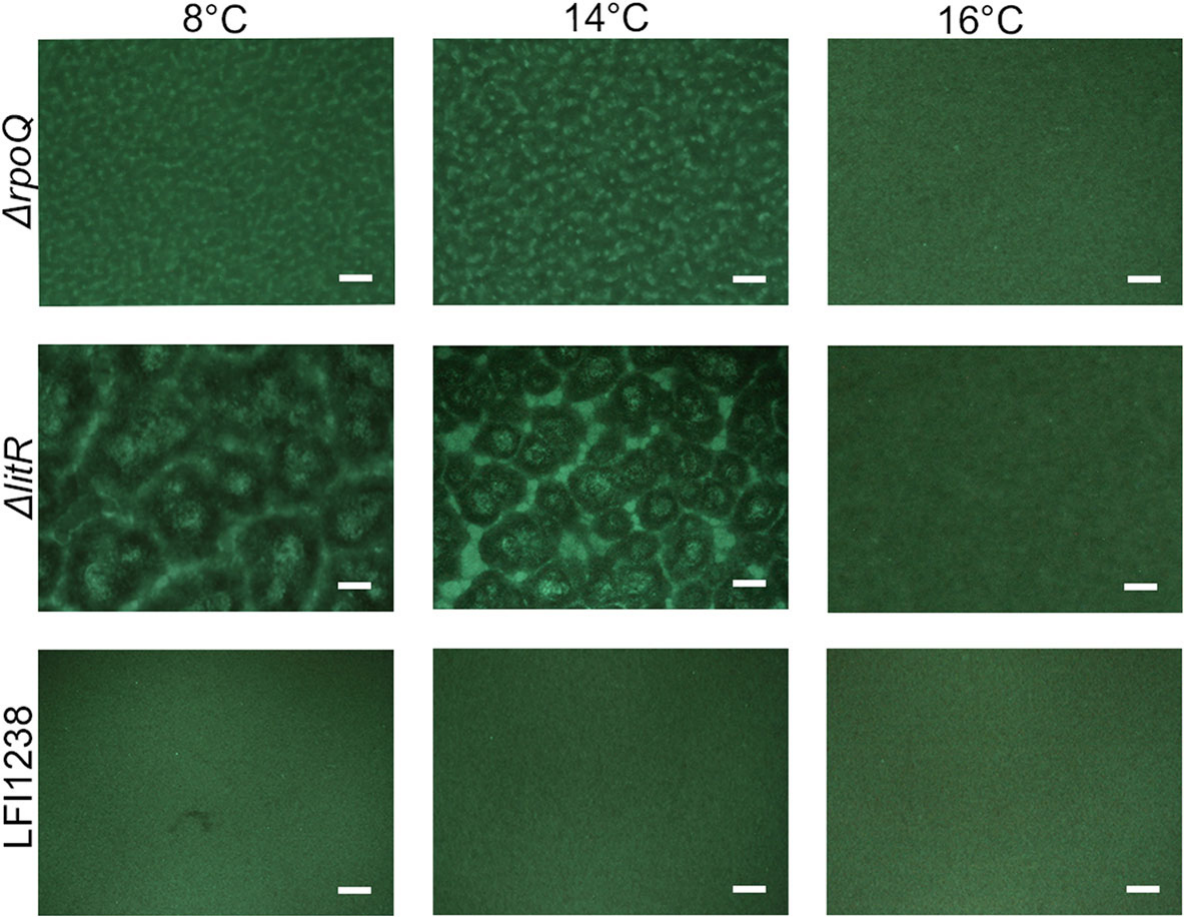
Biofilm formation of GFP-tagged Δ*rpoQ, ΔlitR* and LFI1238 at different temperatures. The GFP tagged strains (LFI1238-pVSV102, Δ*rpoQ-*pVSV102 and Δ*litR-*pVSV102) were allowed to form biofilms in SWT media at 8, 14 and 16 °C. The biofilms were viewed, after 72 h of incubation, in a Nikon Eclipse TS100 microscope at 10× magnification and photographed with Nikon DS-5Mc. Scale bars represent 20 μm

In order to investigate whether *rpoQ* is involved in biofilm formation, the *ΔrpoQ* mutant was allowed to form biofilm in SWT medium at different temperatures using static conditions (Additional file 4: Figure S3). To better visualize the biofilm, GFP-tagged strains were used. The tagged strains were constructed by transferring a constitutive GFP expressing plasmid (pVSV102) into the different mutants and the wild-type strain. As shown in Fig. 2, *ΔrpoQ* produced a biofilm at 8 and 14 °C, which could be clearly visualized after 72 h. Little or no biofilm was observed at 16 °C for the different strains. The biofilm produced by the *ΔrpoQ* mutant does not show large mushroom shaped structures similar to those produced by *ΔlitR* (Fig. 2 and [31]); instead the *ΔrpoQ* mutant formed a more regular and flat biofilm with smaller micro-colonies and structures. Above the microscopically visual *ΔrpoQ* biofilm structures is a thick and slimy extracellular matrix without or with few embedded bacteria (Additional file 5: Figure S4). The complementary strain *ΔrpoQc* behaved similar to the wild-type, whereas the double mutant *ΔlitR-rpoQ*^*−*^ produced a biofilm with mushroom structure similar to the one produced by the *ΔlitR* mutant (Additional file 4: Figure S3).

### RpoQ regulates motility in *A. salmonicida*

**Fig. 3:**
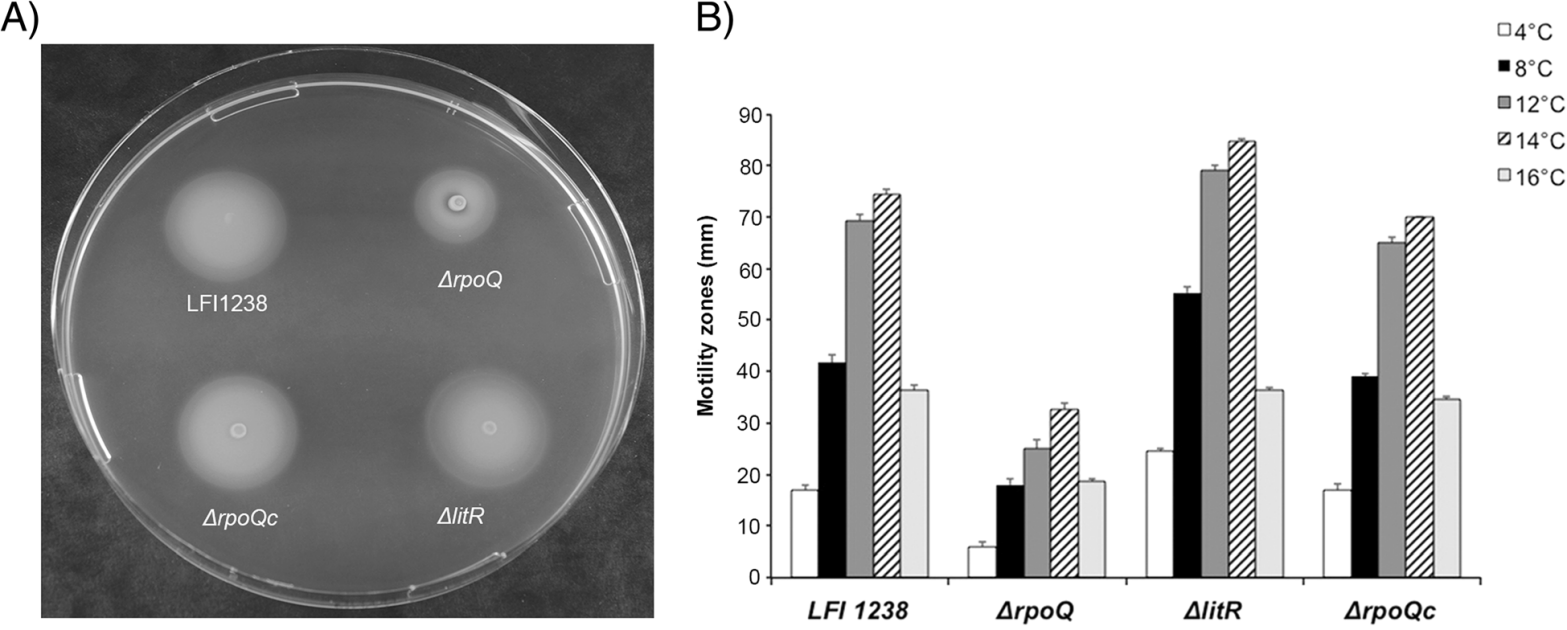
Motility of LFI1238, Δ*rpoQ, ΔrpoQ*_*c*_ and Δ*litR* at different temperatures*.* **a** Soft agar plate showing motility zones of LFI1238, Δ*rpoQ, ΔrpoQ*_***c***_ and Δ*litR* after 5 days incubation at 8 °C. **b** Motility zones (mm) of LFI1238, Δ*rpoQ, ΔlitR,* and Δ*rpoQ*_*c*_ measured after 5 days incubation at different temperatures (4–16 °C). The error bars present the standard deviation of biological triplicate

The flagellum is required for motility of bacteria, mediating their movements towards favorable environments or away from harmful conditions [41, 42]. Previous studies have shown that *A. salmonicida* is more motile at 12 °C than at 4 °C, and that LitR is a negative regulator of motility [22]. Here we analyzed the influence of RpoQ on the motility of *A. salmonicida* at different temperatures (4 to 16 °C). Deletion of *rpoQ* resulted in a strain with reduced motility compared to the wild-type and the *ΔlitR* mutant at all tested temperatures (Fig. 3 and Additional file 6: Table S2). After 5 days of incubation at 4 °C the *ΔrpoQ* mutant was almost non-motile and the motility zone was only 6.0 ± 1.0 mm. At higher temperatures (8 to 16 °C) the motility of the *ΔrpoQ* mutant was between 36 and 51% compared to the motility of wild-type. Hence, the incubation temperature did not seem to affect the regulatory effect of RpoQ on the motility. Similar to the wild-type and *ΔlitR,* the *ΔrpoQ* mutant shows highest motility at 14 °C. The *ΔrpoQc* behaved similar to the wild-type (Fig. 3a and b).

### RpoQ is a negative regulator of biofilm

RpoQ is believed to function downstream of LitR in the QS cascade in *A. salmonicida* [31], and as shown above, deletion of *rpoQ* resulted in increased biofilm formation (Fig. 2). Hence, it was of interest to examine the influence of overexpressing *rpoQ* on the *ΔlitR* biofilm formation. For this purpose, the control vector (pTM214) and the inducible *rpoQ* vector (pTM214-*rpoQ*) were separately transferred to the *ΔlitR* mutant strain and the wild-type LFI1238 by conjugation. The biofilm assay was performed as before in SWT medium (4 to 16 °C) but with 1 mM IPTG to induce expression of *rpoQ*.

**Fig. 4:**
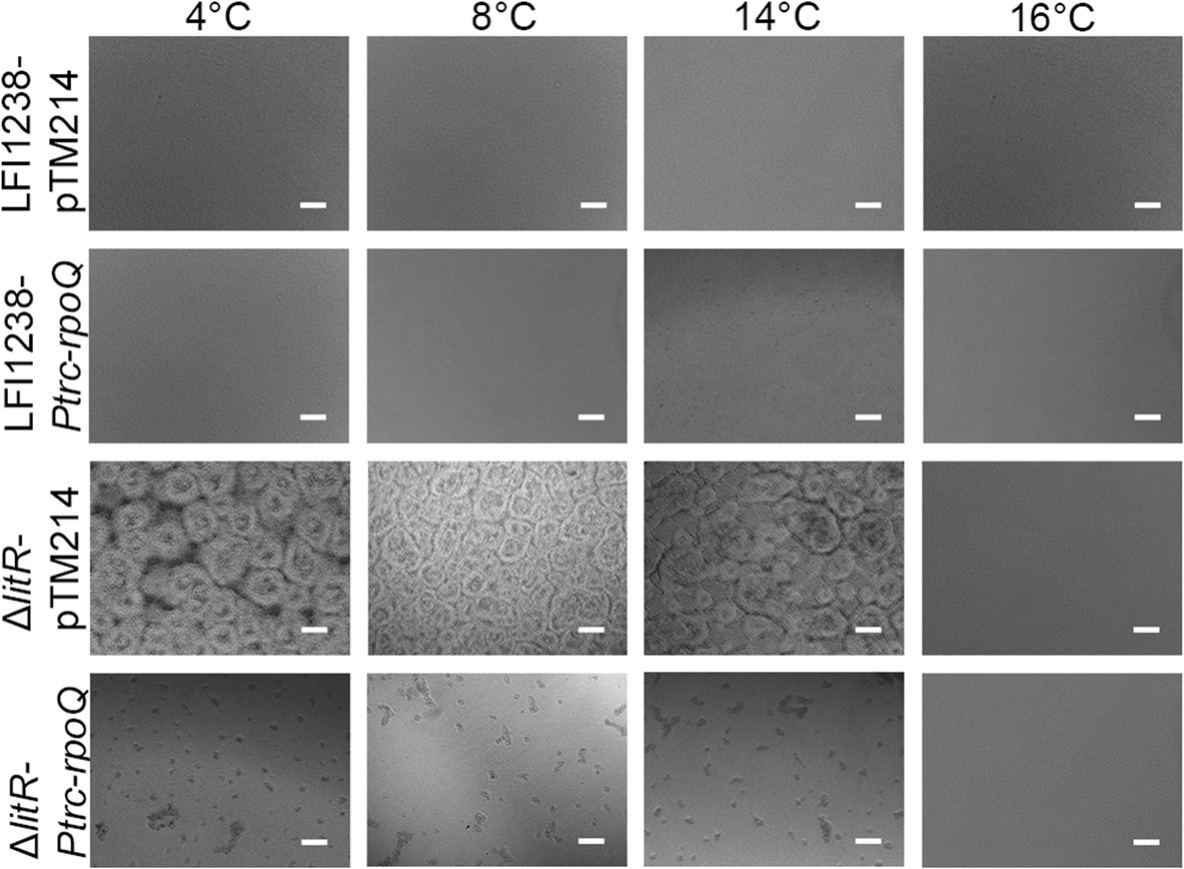
The effect of the RpoQ on the biofilm formation of LFI1238 and Δ*litR.* The biofilms of LFI1238 and Δ*litR* harboring the pTM214 (control vector) and LFI1238 and Δ*litR* harboring the *P*_*trc*_*-rpoQ* (*rpoQ* overexpression vector) were allowed to form in SWT medium supplemented with 1 mM IPTG. The biofilms were incubated for 72 h at different temperatures (4 to 16 °C). The biofilms were viewed in Nikon Eclipse TS100 microscope at 10× magnification and photographed with Nikon DS-5Mc. Scale bars represent 20 μm

As shown in Fig. 4, overexpression of *rpoQ* disrupted or inhibited the biofilm formation produced by *ΔlitR* (*ΔlitR-Ptrc-rpoQ* at 4 to 14 °C) leaving small aggregates in the wells*,* whereas the *ΔlitR* biofilm formation was unaffected by the presence of the control vector (*ΔlitR-*pTM214) at all temperatures. Biofilm formation does not occur at 16 °C, and hence no effects of the overproduced *rpoQ* was observed. Neither was any changes observed when *rpoQ* was overexpressed in wild-type cells (LFI1238-*Ptrc-rpoQ*) (Fig. 4).

### Overexpression of RpoQ decreases motility in *A. salmonicida*

**Fig. 5:**
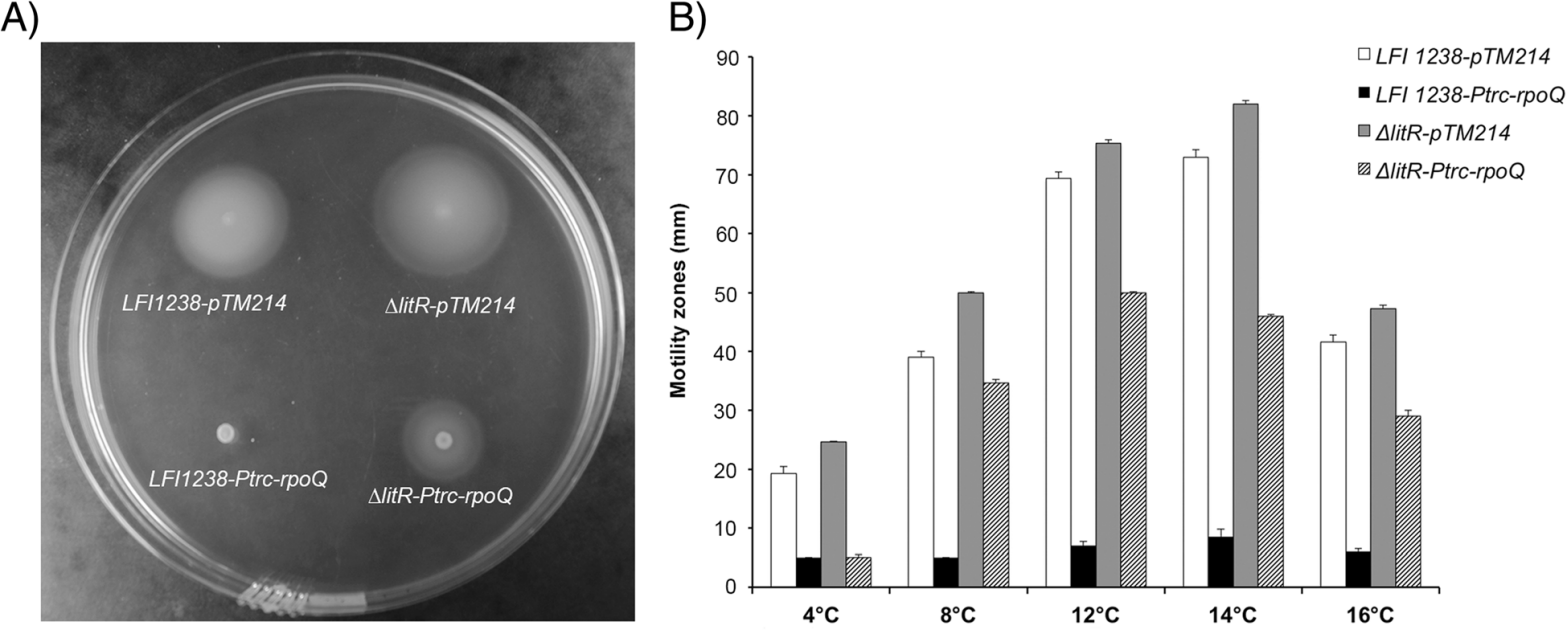
Motility assay on soft agar supplemented with 1 mM IPTG. **a** Soft agar plate showing motility zones of LFI1238 and Δ*litR* harboring the pTM214 (control vector) and LFI1238 and Δ*litR* harboring the *P*_*trc*_*-rpoQ* (*rpoQ* overexpression vector) at 8 °C after 5 days. **b** Motility zones (mm) of LFI1238-pTM214, Δ*litR-*pTM214, LFI1238-*P*_*trc*_*-rpoQ* and Δ*litR-P*_*trc*_*-rpoQ* after 5 days of incubation at temperatures ranging from 4 to 16 °C. The error bars present the standard deviation of biological triplicate

In the experiments performed above we show that *rpoQ* is required for full wild- type motility at all temperatures (Fig. 3) and that overexpression of *rpoQ* has a negative effect on the biofilm forming ability of the *ΔlitR* mutant (Fig. 4). It therefore was of interest to analyze if overexpressed *rpoQ* also affected the motility of the wild-type and the *ΔlitR* mutant. As shown in Fig. 5, overexpression of *rpoQ* repressed the motility in both strains. Most notable, overexpression of *rpoQ* in the wild-type resulted in a completely non-motile strain when incubated at 4 and 8 °C, and the size of the spotted LFI1238*-Ptrc-rpoQ* colony (5 mm) did not change at any of the two temperatures during the 5 days of the experiment (Fig. 5a and b). At 12, 14 and 16 °C small motility zones (7–9 mm) were observed for LFI1238-*Ptrc-rpoQ* showing that overexpression of *rpoQ* in the wild-type does not result in complete shutdown of the motility at these temperatures. Overexpression of *rpoQ* in the *ΔlitR* also resulted in clearly diminished motility zones at all temperatures (Fig. 5b and Additional file 7: Table S3).

## Discussion

Bacteria continually face changes in their environment such as temperature fluctuations, nutrient accessibility and pH changes. In order to adapt to these changes and often challenging conditions, bacteria have developed various responses. Alternative sigma factors such as RpoS provide a main line of responses to changes in the environment by altering gene transcription [43, 44]. Several studies have shown a connection between RpoS and QS in different vibrios [38, 45–48]. When Cao et al. (2012) described the alternative sigma factor RpoQ in *A. fischeri* a homologue was only found in *A. salmonicida* [28]. However, since then the genomes of *A. wodanis* [49] and *A. logei* (*A.logei* S5–186 GeneBank accession no. AJY02000108.1) have become available. Analysis show that they also encode an RpoQ homolog with four conserved domains (σ^70^ regions 1–4). RpoQ of *A. salmonicida* shares a high amino acid sequence identity (99%) with its homolog in *A. logei* whereas the amino acid sequence identity is 72% with *A. fischeri* and 69% with *A. wodanis.* Region 2 and region 4 of the putative RpoQ are well conserved between the four species, whereas region 3 is less conserved (Additional file 8: Figure S5).

RpoQ is regulated by LuxO through LitR in *A. fischeri* [28]. Similarly, our previous microarray results suggested that LitR is a positive regulator of RpoQ in *A. salmonicida* [31]. In the study presented here, we show that RpoQ is involved in regulation of colony morphology, adhesion, biofilm and motility similar to LitR. However, since RpoQ is suspected to act downstream of the master regulator LitR, one can expect that the *ΔlitR* mutant expresses phenotypes that are independent of RpoQ regulation.

The *ΔrpoQ* mutant demonstrated a stronger and an earlier onset of the rugose colony morphology as compared to the *ΔlitR* mutant. A rugose colony phenotype usually develops when the bacteria produce high amounts of polysaccharides, suggesting that more polysaccharides are made by the *ΔrpoQ* mutant. We know from our previous work that LitR represses the expression of the *symbiosis polysaccharide* (*syp*) operon, and that inactivation of *syp* (*sypC, sypP* and *sypQ*) in the *ΔlitR* mutant results in smooth colonies [31]. Hence, it is likely that LitR performs its activity on *syp* through RpoQ and that activation of RpoQ leads to a strong(er) repression of *syp*. The weaker rugose colony morphology of the *ΔlitR* mutant may be due to low levels of LitR-independent *rpoQ* expression, consistent with our previous microarray results that show expression of *rpoQ* in the *ΔlitR* mutant [31]. Hence, some repression of *syp* via RpoQ probably occurs in the *ΔlitR* mutant. Whereas in the *ΔrpoQ* mutant there is zero expression of *rpoQ* resulting in no or low *syp* repression and stronger rugosity*.*

**Fig. 6:**
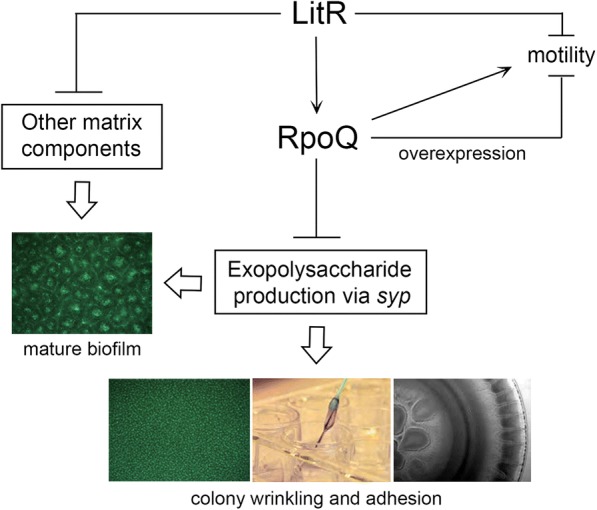
Proposed model for regulation of QS related phenotypes in *A. salmonicida*. At high cell densities, LitR is produced in response to AHLs and acts as a positive regulator of *rpoQ* expression. LitR, probably via RpoQ, downregulates motility and expression of exopolysaccharides. The Δ*litR* mutant shows a mature biofilm with mushroom shaped structures, whereas the Δ*rpoQ* biofilm is more flat and regular. Thus, in addition to repression of exopolysaccharides via RpoQ, LitR represses other biofilm matrix components independent of RpoQ that are required for building mature mushroom structures (e.g. lipoproteins, protein filaments). Therefore, at high cell densities both RpoQ dependent and independent processes are needed for down regulation of the mature biofilm. The Δ*rpoQ* mutant shows decreased motility suggesting that RpoQ may also act as a positive activator of motility. Arrows and lines with bar ends indicate pathways of positive and negative regulation, respectively, and may consist of several steps. The thicker, empty arrows indicate the resulting phenotypes

Both LitR and RpoQ are negative regulators of biofilm formation in *A. salmonicida*. However, the *ΔrpoQ* mutant formed a biofilm morphologically different from the *ΔlitR* mutant. The biofilm produced by the *ΔrpoQ* was less mature and relatively flat and compact, without the large mushroom structures exhibited by the *ΔlitR* mutant. Additionally, the biofilm produced by *ΔrpoQ* contained a heavy and slimy extracellular matrix substance above the biofilm cells attached to the substratum (Additional file 5: Figure S4). This slimy matrix is likely due to high amounts of polysaccharides (i.g. *syp* expression as discussed above) that are common components of the extracellular matrix of biofilms, together with proteins and eDNA [50]. When we previously analyzed the *ΔlitR* biofilm we found that major components were polysaccharides and proteins, and by using electron microscopy we were able to see a network of fibers that connected biofilm cells together. The microarray analysis identified, in addition to *syp*, some lipoprotein, pili, flagella, and curli genes that were upregulated in the *ΔlitR* mutant [31]. Hence, LitR may repress some lipoproteins or filament structures needed to build up this mushroom-shaped biofilm architecture. Thus, one explanation for the observed biofilm morphology of the *ΔrpoQ* mutant may be that polysaccharide production is obtained through expression of *syp*, whereas expression of a functional LitR down-regulates genes involved in building mushroom shaped structures. When we inactivated *syp* in the *ΔlitR* mutant we found that although the rugose colony morphology reverted to wild-type morphology (smooth), some biofilm formation still occurred when using SWT medium [31]. Indeed, the biofilms produced by the *ΔlitRsyp*^*−*^ mutants resembles the biofilm produced by *ΔrpoQ* but without the slimy extracellular matrix. We therefore believe that the pathway through which LitR represses genes responsible for building the mushroom-shaped structures is different from the pathway through which LitR represses *syp* (via RpoQ) resulting in rugose colony morphology (Fig. 6).

Both mutants produce biofilms that are loosely attached; however, in contrast to the *ΔlitR* biofilm, the *ΔrpoQ* biofilm is not able to withstand the washing steps required after staining with crystal violet. To our knowledge RpoQ, has not been shown to be involved in biofilm formation of *A. fischeri* or any other aliivibrios. However, studies have shown that RpoS is able to enhance or repress biofilm formation in *E. coli* and other bacteria [51–53]. Additionally, RpoS has been shown to be involved in cell attachment and the maturation of biofilm [30, 54, 55], and inactivation RpoX in *V. alginolyticus* results in cells with decreased ability to form biofilm [29]. Likewise, inactivation of *rpoQ* in *A. salmonicida* may have reduced the ability of the bacteria to attach to the abiotic surface and to build a mature biofilm. Another explanation is that the *ΔrpoQ* biofilm contains a higher amount of a heavy, extracellular, slimy polysaccharide matrix that tears the biofilm away from the substratum when the medium or wash solutions is being poured out or a combination of both.

Thus, as shown in Fig. 6 we propose that RpoQ and LitR function in the same pathway, where RpoQ functions downstream of the LitR and is involved in repression of biofilm and the wrinkled colony morphology in *A. salmonicida*. The negative regulation cascade of extracellular polysaccharide matrix from LitR to the *syp* operon is probably operated through RpoQ, either directly or indirectly. The phenotypes are likely regulated in a cell density manner as previously discussed, where the development of a mushroom shaped biofilm structures and wrinkled colony morphology are initiated when neither AinS or LuxI AHLs are present at low cell density [19, 22, 31]. At high cell density when AHLs are produced, LitR represses genes required for building a mature biofilm structure, and activates *rpoQ* leading to repression of *syp*.

Inactivation of either *rpoQ* or *litR* had the opposite effect on motility in *A. salmonicida*. Unlike the *ΔlitR* mutant, which is more motile than the wild-type strain*,* the *ΔrpoQ* mutant exhibited significantly reduced motility. The complementary strain *ΔrpoQc* showed wild-type motility, suggesting that the termination of motility is due to *rpoQ* deletion and not to other factors. Reduced motility due to disruption of sigma factors has been reported for other bacteria, and inactivation of *rpoS* in *Y. pseudotuberculosis* results in decreased motility due to downregulation of the flagella master regulatory gene *flhDC* [51]. Thus, RpoQ may work in a similar manner by altering transcription of genes responsible for flagellar assembly or flagellar biosynthesis in *A. salmonicida*. Flagellum-mediated motility is important for specific stages of biofilm formation and surface attachment in several bacteria [56–58], and disruption of flagella biosynthesis is known to decrease attachment and alter biofilm architecture [59–62]. For example, loss of motility in *E. coli* affected the biofilm architecture, where poorly motile strains formed flatter biofilms compared to highly motile strains, which displayed more mature vertical biofilm structures [63]. Thus, it is tempting to speculate that the decreased motility of the *ΔrpoQ* mutant resulted in cells with reduced ability to attach and form mature biofilms.

Furthermore, overexpression of *rpoQ* resulted in non-motile wild-type cells and *ΔlitR* cells with reduced motility. These results are similar to those obtained with *A. fischeri*, where the overexpression of *rpoQ* in the wild-type and *ΔlitR* mutant resulted in non-motile strains [28]. The finding that both deletion and overexpression of *rpoQ* in *A. salmonicida* resulted in bacteria with reduced motility is interesting, but at the same time difficult to interpret. We know that RpoQ functions downstream of LitR and that LitR is a negative regulator of motility at high cell density [22, 31]. Thus, we may have expected to observe a similar effect on motility when we knocked out *rpoQ*. However, the *ΔrpoQ* mutant show decreased motility compared to the wild-type indicating that RpoQ is a positive regulator of motility (Fig. 6). This may suggest that at low cell densities some *litR* independent expression of *rpoQ* occurs and that RpoQ activates genes involved in flagellar biosynthesis. As the cell population increases *litR* will be expressed leading to increased levels of RpoQ. High RpoQ levels (overexpression of *rpoQ*) then turns down motility probably by acting as an activator of genes involved in down regulation of the flagellar apparatus. Hence, RpoQ probably controls genes responsible for both promoting or repressing motility depending on growth phase, environmental conditions and stress factors. Our results show that regulation of motility in *A. salmonicida* is complex similar to other vibrios [64] and probably involves several regulatory genes and factors, which is still unrevealed.

Temperature is an important factor in developing cold-water vibriosis and for production of AHLs in *A. salmonicida.* When the bacteria is grown at temperature above the disease limit (16 °C), the production of AHLs is nearly absent [19]*.* Our results from the biofilm and colony morphology assays show that the *ΔrpoQ* mutant behaves as the wild-type strain and the *ΔlitR* mutant when the assays are performed at 16 °C, and neither of the strains forms rugose colonies or biofilm at this temperature. This shows that RpoQ, similar to LitR, represses formation of biofilm and rugose colonies more at low temperatures (4–14 °C), and at 16 °C the effect of the *rpoQ* deletion is absent with regard to these phenotypes. Interestingly, this temperature effect was not observed when the motility of the *ΔrpoQ* mutant was analyzed, and at 16 °C the motility of the *ΔrpoQ* mutant was still clearly reduced compared to the wild-type. This implies that RpoQ is expressed and is able to regulate motility in *A. salmonicida* at temperatures above the limit for developing cold water vibriosis, and at conditions when AHL concentrations are expected to be low.

## Conclusion

In this work we have shown that the alternative sigma factor RpoQ regulates motility, colony morphology and biofilm formation in *A. salmonicida*. This broad range of different phenotypes suggests that RpoQ is involved in a regulatory hierarchy influencing expression of a large panel of genes. Overexpression of RpoQ led to disruption of the biofilm produced by *ΔlitR,* paralyzed the motility of the wild-type *A. salmonicida* and caused a reduction in *ΔlitR* motility*.* These findings confirm that the RpoQ is a novel factor in the QS and functions downstream of the LitR. However, further studies are needed to understand exactly how LitR and RpoQ work together or independently to regulate the QS dependent phenotypes investigated here, and to identify genes regulated by RpoQ.

## Additional files

Additional file 1: **Figure S1.** The figure shows growth curves of *A. salmonicida* wild type and *rpoQ* mutants. (DOCX 126 kb)

Additional file 2: **Figure S2.** The figure shows colony morphology of *ΔlitR* after 3 weeks of incubation. (DOCX 101 kb)

Additional file 3: **Table S1.** The table lists grading of adherence of *A. salmonicida* wild-type and mutants on SWT agar. (DOCX 16 kb)

Additional file 4: **Figure S3.** The figure shows biofilm formation of *A. salmonicida* wild-type LFI1238 and mutants. (DOCX 642 kb)

Additional file 5: **Figure S4.** The figure shows the slimy extracellular matrix formed by *ΔrpoQ* in the biofilm assay. (DOCX 701 kb)

Additional file 6: **Table S2.** The table lists motility zones of LFI1238, *ΔrpoQ*, *ΔrpoQc* and *ΔlitR* formed on soft agar plates. (DOCX 15 kb)

Additional file 7: **Table S3.** The table lists motility zones formed on soft agar plates supplemented with 1 mM IPTG. (DOCX 16 kb)

Additional file 8: **Figure S5.** The figure shows alignment and phylogeny of RpoQ, RpoS and RpoX. (DOCX 699 kb)

## Abbreviations

IPTG: Isopropyl β-D-1-thiogalactopyranoside
min: Minutes
OD_600_: Optical density measured at 600 nm
ON: Overnight
PCR: Polymerase chain reaction
QS: Quorum sensing
rpm: Rounds per minute
