# Supplementary material for: The alternative sigma factor RpoQ regulates colony morphology, biofilm formation and motility in the fish pathogen Aliivibrio salmonicida
Source: BMC Microbiol. 2018 Sep 12;18:116. doi: 10.1186/s12866-018-1258-9 (PMC6134601; doi:10.1186/s12866-018-1258-9)
Supplement: Supplementary file 1 — Figure S1. The figure shows growth curves of A. salmonicida wild type and rpoQ mutants. (DOCX 126 kb) [file 12866_2018_1258_MOESM1_ESM.docx]

Additional file 1


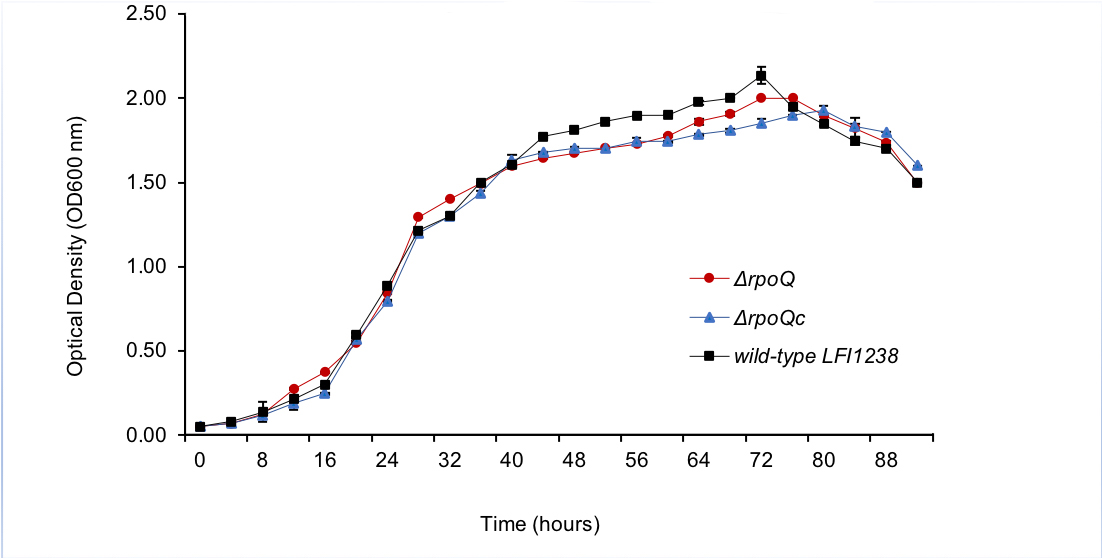


Figure S1. **Growth curves of** *Δ****rpoQ,*** *Δ****rpoQc* and wild-type LFI1238.** The bacterial cultures were grown in SWT medium for 92 hours at 8°C and 200 rpm. The bacterial cultures were diluted to a starting OD_600_ of 0.05. The OD_600_ measurements were performed at 4 hours intervals. The error bars represent the standard deviation of biological triplicates.
