# Supplementary material for: The alternative sigma factor RpoQ regulates colony morphology, biofilm formation and motility in the fish pathogen Aliivibrio salmonicida
Source: BMC Microbiol. 2018 Sep 12;18:116. doi: 10.1186/s12866-018-1258-9 (PMC6134601; doi:10.1186/s12866-018-1258-9)
Supplement: Supplementary file 2 — Figure S2. The figure shows colony morphology of ΔlitR after 3 weeks of incubation. (DOCX 101 kb) [file 12866_2018_1258_MOESM2_ESM.docx]

Additional file 2


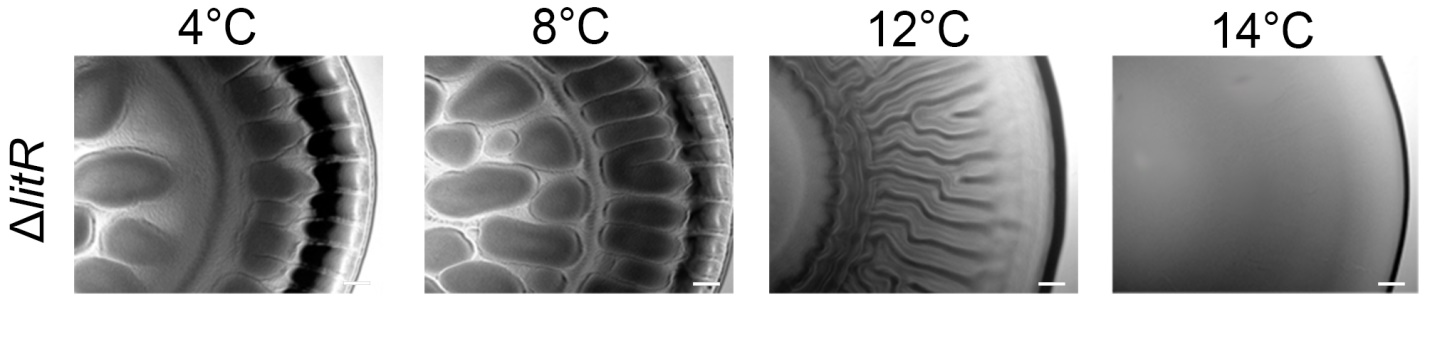


Figure S2. **Colony morphology of** *Δ****litR* at different temperatures.** The colonies were allowed to form on SWT plates for 3 weeks at 4, 8, 12 and 14°C. The colonies were viewed in a Zeiss Primo Vert microscope at 4x magnification. Scale bars present 0.5mm.
