# Supplementary material for: The alternative sigma factor RpoQ regulates colony morphology, biofilm formation and motility in the fish pathogen Aliivibrio salmonicida
Source: BMC Microbiol. 2018 Sep 12;18:116. doi: 10.1186/s12866-018-1258-9 (PMC6134601; doi:10.1186/s12866-018-1258-9)
Supplement: Supplementary file 3 — Table S1. The table lists grading of adherence of A. salmonicida wild-type and mutants on SWT agar. (DOCX 16 kb) [file 12866_2018_1258_MOESM3_ESM.docx]

Additional file 3

Table S1. **Grading of** **adherence of LFI1238,** *Δ****rpoQ,*** *Δ****rpoQ_c_* and** *Δ****litR*** **to SWT agar.** The adherence of the colonies was analyzed after 3 weeks incubation at the different temperatures.

| **Bacterial strains** | **4°C** | **8°C** | **12°C** | **14°C** |
| --- | --- | --- | --- | --- |
| LFI1238 | none | none | none | none |
| *ΔrpoQ* | strong | strong | weak | none |
| *ΔlitR* | strong | strong | weak | none |
| *ΔrpoQ_c_* | none | none | none | none |
