# Supplementary material for: The alternative sigma factor RpoQ regulates colony morphology, biofilm formation and motility in the fish pathogen Aliivibrio salmonicida
Source: BMC Microbiol. 2018 Sep 12;18:116. doi: 10.1186/s12866-018-1258-9 (PMC6134601; doi:10.1186/s12866-018-1258-9)
Supplement: Supplementary file 4 — Figure S3. The figure shows biofilm formation of A. salmonicida wild-type LFI1238 and mutants. (DOCX 642 kb) [file 12866_2018_1258_MOESM4_ESM.docx]

Additional file 4


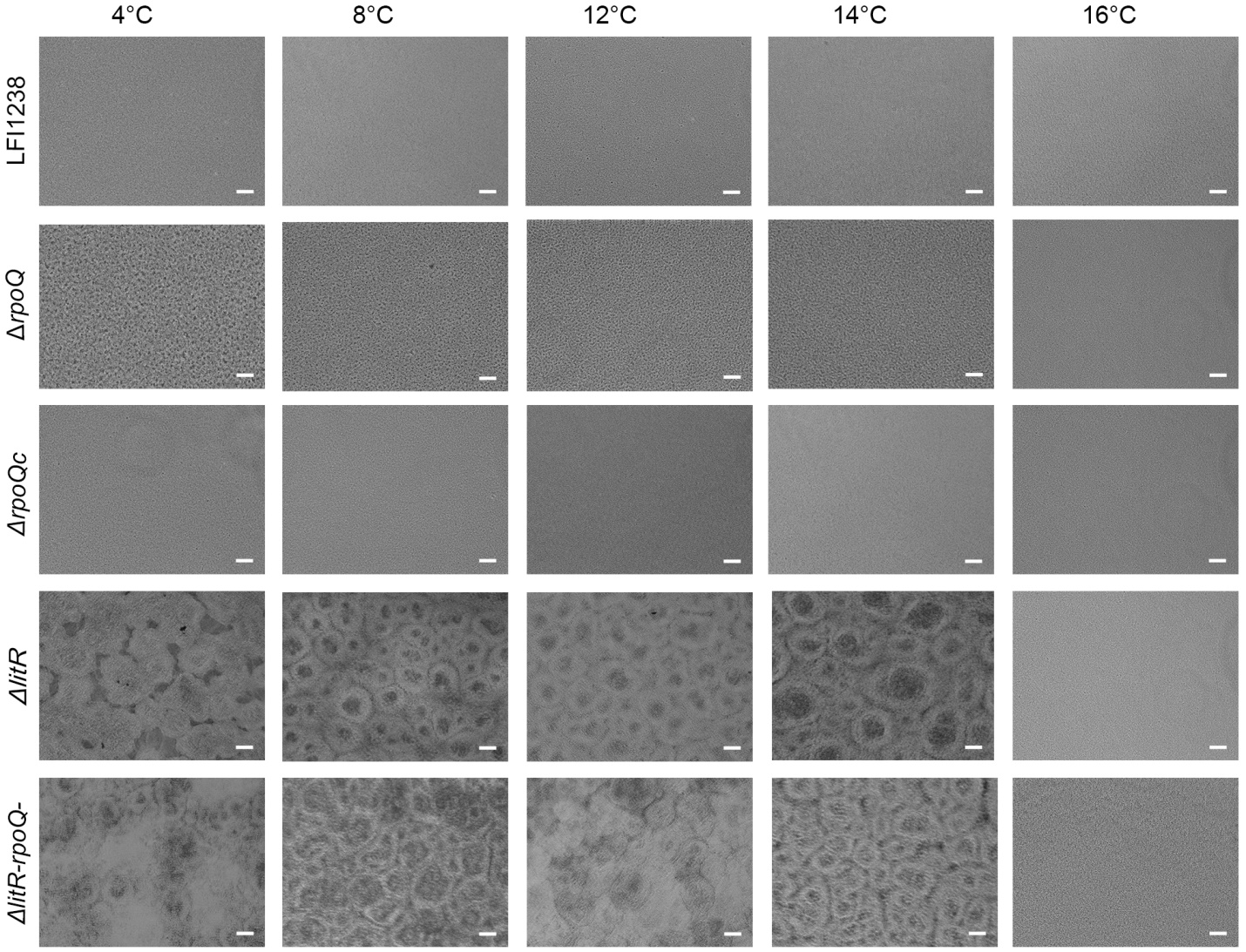


Figure S3. **Biofilm formation of *A. salmonicida* wild-type LFI1238 and mutants.** The different strains (LFI1238, *ΔrpoQ, ΔrpoQc, ΔlitR* and *ΔlitR-rpoQ^-^*) were incubated and allowed to form biofilms in SWT medium at different temperatures (4-16°C). The biofilms formed after 72 hours of incubation were viewed in a Nikon Eclipse TS100 microscope at x10 magnification and photographed by Nikon DS-5Mc. Scale bars present 20μm.
