# Supplementary material for: The alternative sigma factor RpoQ regulates colony morphology, biofilm formation and motility in the fish pathogen Aliivibrio salmonicida
Source: BMC Microbiol. 2018 Sep 12;18:116. doi: 10.1186/s12866-018-1258-9 (PMC6134601; doi:10.1186/s12866-018-1258-9)
Supplement: Supplementary file 5 — Figure S4. The figure shows the slimy extracellular matrix formed by ΔrpoQ in the biofilm assay. (DOCX 701 kb) [file 12866_2018_1258_MOESM5_ESM.docx]

Additional file 5


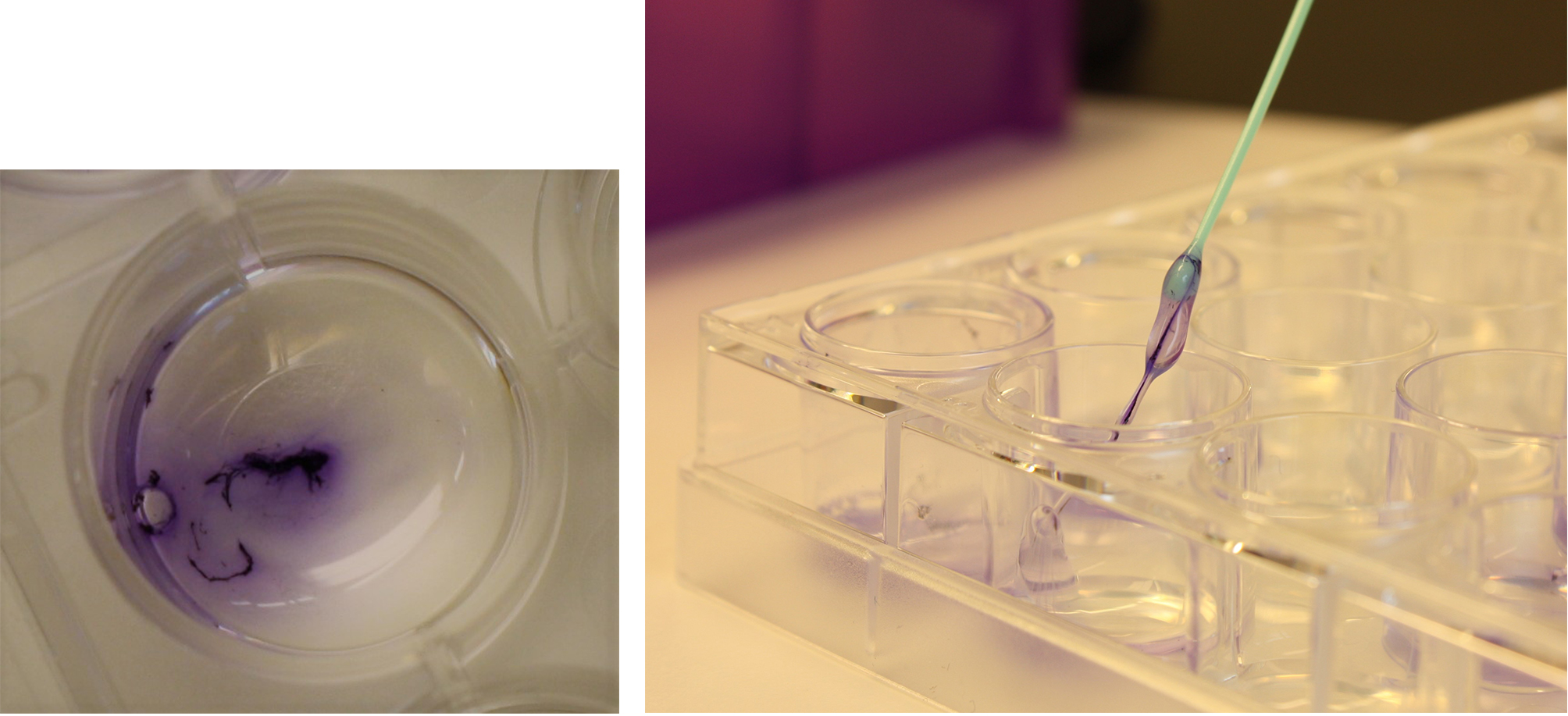


Figure S4. **The extracellular slimy substance of the *ΔrpoQ* biofilm.** *ΔrpoQ* was allowed to form biofilm in SWT medium. After 72 hours incubation a small amount of crystal violet was added into the well to improve the visualization. The picture shows the slimy substance lifted from the well using a plastic-loop. Images were photographed using a Canon camera.
