# Supplementary material for: The alternative sigma factor RpoQ regulates colony morphology, biofilm formation and motility in the fish pathogen Aliivibrio salmonicida
Source: BMC Microbiol. 2018 Sep 12;18:116. doi: 10.1186/s12866-018-1258-9 (PMC6134601; doi:10.1186/s12866-018-1258-9)
Supplement: Supplementary file 6 — Table S2. The table lists motility zones of LFI1238, ΔrpoQ, ΔrpoQc and ΔlitR formed on soft agar plates. (DOCX 15 kb) [file 12866_2018_1258_MOESM6_ESM.docx]

Additional file 6

Table S2. **Motility zones of LFI1238,** *Δ****rpoQ,*** *Δ****rpoQ_c_* and** *Δ****litR*** **formed** **on soft agar plates.** Each value represents the average (mm) of biological triplicates ± standard deviation.

| **Bacterial strains** | **4°C** | **8°C** | **12°C** | **14°C** | **16°C** |
| --- | --- | --- | --- | --- | --- |
| LFI1238 | 17.0 ± 1.0 | 41.6 ± 1.5 | 69.3 ± 1.1 | 74.3 ± 1.1 | 36.3 ± 1.1 |
| *ΔrpoQ* | 6.0 ± 1.0 | 18.0 ± 1.3 | 25.0 ± 1.7 | 32.6 ± 1.1 | 18.6 ± 0.6 |
| *ΔlitR* | 24.6 ± 0.6 | 55.3 ± 1.1 | 79.0 ± 1.0 | 84.6 ± 0.6 | 36.3 ± 0.6 |
| *ΔrpoQ_c_* | 17.0 ± 1.1 | 39.0 ± 0.5 | 65.0 ± 1.0 | 70.0 ± 0.0 | 34.6 ± 0.6 |

* The original size of the spotted colony was 5.0 mm.
