# Supplementary material for: The alternative sigma factor RpoQ regulates colony morphology, biofilm formation and motility in the fish pathogen Aliivibrio salmonicida
Source: BMC Microbiol. 2018 Sep 12;18:116. doi: 10.1186/s12866-018-1258-9 (PMC6134601; doi:10.1186/s12866-018-1258-9)
Supplement: Supplementary file 7 — Table S3. The table lists motility zones formed on soft agar plates supplemented with 1 mM IPTG. (DOCX 16 kb) [file 12866_2018_1258_MOESM7_ESM.docx]

Additional file 7

Table S3. **Motility zones formed on soft agar plates supplemented with 1mM IPTG.** The values represent the average (mm) of biological triplicates ± standard deviation*

| **Bacterial strains** | **4°C** | **8°C** | **12°C** | **14°C** | **16°C** |
| --- | --- | --- | --- | --- | --- |
| *LFI1238*-pTM214 | 19.3 ± 1.1 | 39.0 ± 1.0 | 69.3 ± 1.1 | 73 ± 1.2 | 41.6 ± 1.1 |
| *LFI1238-Ptrc-rpoQ* | 5.0 ± 0.0 | 5.0 ± 0.0 | 7.0 ± 0.5 | 7.4 ± 1.3 | 6.0 ± 0.6 |
| *ΔlitR*-pTM214 | 24.6 ± 0.0 | 50.0 ± 0.2 | 75.3 ± 0.5 | 82.0 ± 0.6 | 47.3 ± 0.6 |
| *ΔlitR-Ptrc-rpoQ* | 5.0 ± 0.0 | 34.6 ± 0.6 | 50.0 ± 0.1 | 46.0 ± 0.2 | 30.0 ± 1.0 |

* The original size of the spotted colony was 5.0 mm.
