# Supplementary material for: The alternative sigma factor RpoQ regulates colony morphology, biofilm formation and motility in the fish pathogen Aliivibrio salmonicida
Source: BMC Microbiol. 2018 Sep 12;18:116. doi: 10.1186/s12866-018-1258-9 (PMC6134601; doi:10.1186/s12866-018-1258-9)
Supplement: Supplementary file 8 — Figure S5. The figure shows alignment and phylogeny of RpoQ, RpoS and RpoX. (DOCX 699 kb) [file 12866_2018_1258_MOESM8_ESM.docx]

Additional file 8
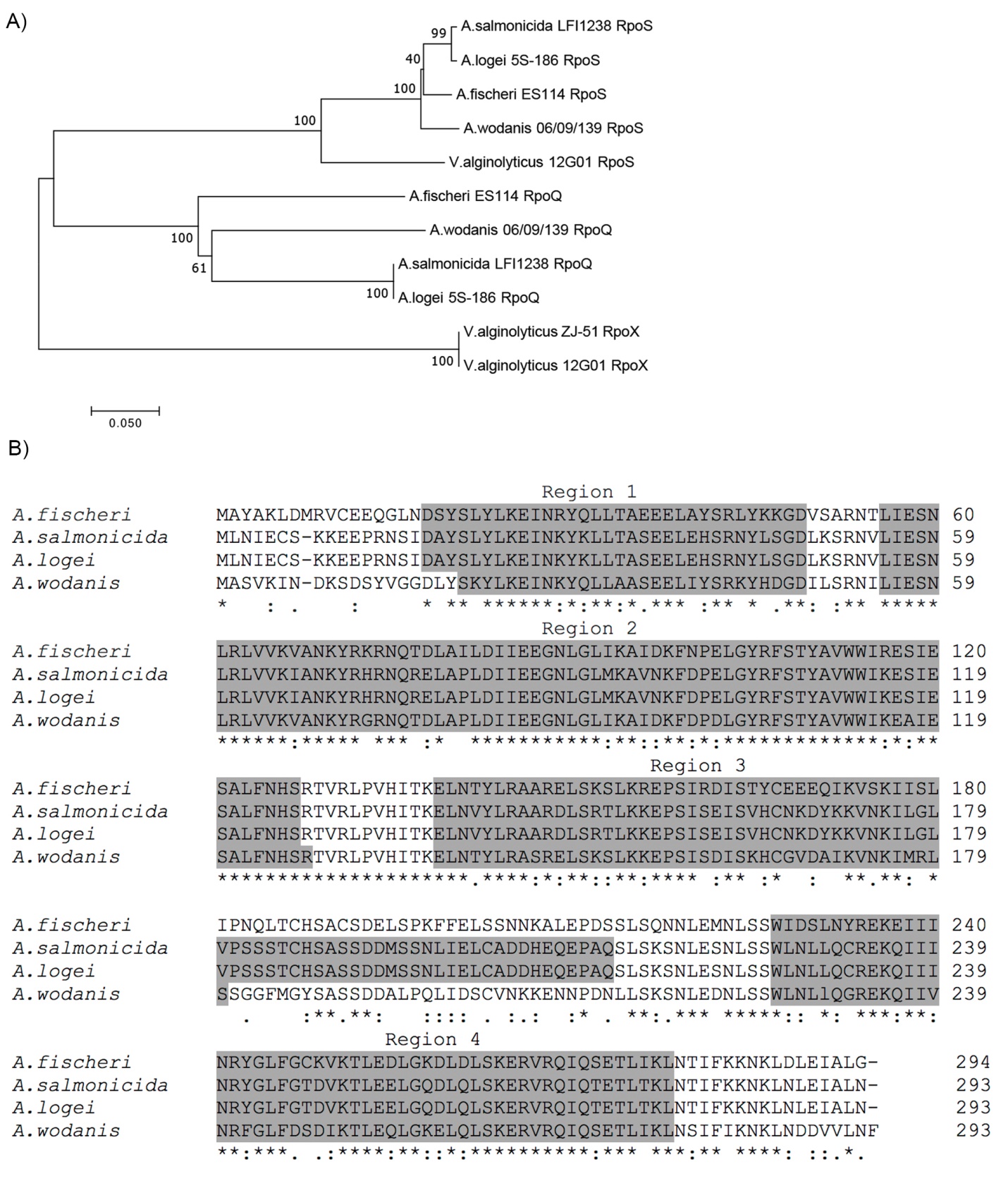


Figure S5. **Alignment and phylogenetic analyses of RpoQ, RpoS and RpoX amino acid sequences. A)** The phylogenetic tree was constructed using neighbor joining and clustalW aligned amino acid sequences of RpoQ from *A. salmonicida* LFI1238 (acc.no. WP_012551679, *VSAL*_*II0319*), *A. fischeri* ES114 (acc.no. YP_206973, *VF*_*A1015*), *A. logei* S5-186 (acc.no. OEF11440, *A1Q5*_*19865*) and *A. wodanis* 06/09/139 (acc.no. CED57794, *AWOD*_*II1179*); RpoS from *A. salmonicida* (acc.no. WP_012550978, *VSAL*_*I2506*), *A. fischeri* ES114 (acc.no. YP_205450, *VF*_*2067*), *A. logei* S5-186 (acc.no. OEF20103, *A1Q5_16955*), *A. wodanis* 06/09/139 (acc.no. CED72209, *AWOD_I_2147*) and *V*. *alginolyticus* 12G01 (acc.no. EAS74640, *V12G01_13719*); and RpoX from *V*. *alginolyticus* ZJ-51 (acc.no. ACJ09227, locus tag not available) and *V*. *alginolyticus* 12G01 (acc.no. ZP_01261551, *V12G01*_*06616*) was included as an outgroup. Numbers shown on the branch points of phylogenic tree represent the bootstrap values (%). The scale bar (0.050) represents amino acid substitutions per site. **B)** ClustalW alignment of RpoQ amino acid sequences from *A. fischeri* ES114, *A. salmonicida* LFI1238, *A. logei* 5S-186 and *A. wodanis* 06/09/136. (⁎) indicates a full conserved residue, (:) indicates a fully conserved strong group and (.) indicates a fully conserved weak group. The four conserved regions (region 1-4) in RpoQ are highlighted in grey color.
